# Driving Pattern Analysis, Gear Shift Classification, and Fuel Efficiency in Light-Duty Vehicles: A Machine Learning Approach Using GPS and OBD II PID Signals

**DOI:** 10.3390/s25134043

**Published:** 2025-06-28

**Authors:** Juan José Molina-Campoverde, Juan Zurita-Jara, Paúl Molina-Campoverde

**Affiliations:** Grupo de Ingeniería Automotriz, Movilidad y Transporte (GiAUTO), Carrera de Ingeniería Automotriz-Campus Sur, Universidad Politécnica Salesiana, Quito 170702, Ecuador; jzuritaj@est.ups.edu.ec (J.Z.-J.); pmolinac1@ups.edu.ec (P.M.-C.)

**Keywords:** GPS, KNN, K-means, OBD II, stopping times, PID, fuel consumption, data logger, longitudinal dynamics

## Abstract

This study proposes an automatic gear shift classification algorithm in M1 category vehicles using data acquired through the onboard diagnostic system (OBD II) and GPS. The proposed approach is based on the analysis of identification parameters (PIDs), such as manifold absolute pressure (MAP), revolutions per minute (RPM), vehicle speed (VSS), torque, power, stall times, and longitudinal dynamics, to determine the efficiency and behavior of the vehicle in each of its gears. In addition, the unsupervised K-means algorithm was implemented to analyze vehicle gear changes, identify driving patterns, and segment the data into meaningful groups. Machine learning techniques, including K-Nearest Neighbors (KNN), decision trees, logistic regression, and Support Vector Machines (SVMs), were employed to classify gear shifts accurately. After a thorough evaluation, the KNN (Fine KNN) model proved to be the most effective, achieving an accuracy of 99.7%, an error rate of 0.3%, a precision of 99.8%, a recall of 99.7%, and an F1-score of 99.8%, outperforming other models in terms of accuracy, robustness, and balance between metrics. A multiple linear regression model was developed to estimate instantaneous fuel consumption (in L/100 km) using the gear predicted by the KNN algorithm and other relevant variables. The model, built on over 66,000 valid observations, achieved an R^2^ of 0.897 and a root mean square error (RMSE) of 2.06, indicating a strong fit. Results showed that higher gears (3, 4, and 5) are associated with lower fuel consumption. In contrast, a neutral gear presented the highest levels of consumption and variability, especially during prolonged idling periods in heavy traffic conditions. In future work, we propose integrating this algorithm into driver assistance systems (ADAS) and exploring its applicability in autonomous vehicles to enhance real-time decision making. Such integration could optimize gear shift timing based on dynamic factors like road conditions, traffic density, and driver behavior, ultimately contributing to improved fuel efficiency and overall vehicle performance.

## 1. Introduction

Vehicular congestion has become a global problem with economic repercussions. It reduces the speed between transfers and it reduces the sustainable development of cities [1,2,3,4]. Moreover, it is a common problem in growing cities due to the increase in the number of vehicles, while the capacity limits of roads are reduced [5,6]. The transport sector should represent an intersection between urban development and environmental care [7]; still, it has become difficult in developing countries such as Ecuador, where urbanization reaches 65% of the population [8,9]. Additionally, drivers with aggressive driving characteristics are responsible for increased vehicular traffic [10,11,12] due to stopping conditions after sudden accelerations.

Despite the progress made in recent years in characterizing driving styles and their correlation with fuel consumption, the influence of variables such as vehicle type and traffic conditions can vary considerably, making this an ongoing research challenge. Recent studies have highlighted the substantial impact of driving behavior on fuel efficiency. The concept of eco-driving incorporates various aspects such as driving style, route selection, and vehicle operating choices [13]. It has been shown that poor traffic conditions can significantly reduce energy efficiency, with the magnitude of the impact varying depending on the type of road [14]. Furthermore, machine learning methods have been applied to predict fuel consumption by analyzing personalized driving behavior and incorporating macroscopic and microscopic traffic parameters [15]. Ma and Wang [16] showed that differences in pedal usage and following distance can lead to fuel consumption deviations of up to 29% for light trucks and 15% for passenger cars under identical traffic conditions.

In the study by Zamaras et al. [17], a micro-traffic model was developed to analyze an urban corridor in Turin, Italy. The model was calibrated using 20% of the driving behavior data collected from the corridor and combined with vehicle energy consumption and emission simulators. Their results indicated that traffic congestion conditions can cause variations in fuel consumption ranging from −25.8% to 20.9% in EURO 5 light vehicles. Similarly, Rodríguez et al. [18] conducted experiments on a fleet of light vehicles in Bogotá, measuring CO, CO_2_, NO_x_, and HC emissions. Their findings showed a strong correlation between these emissions and the vehicles’ specific power demand (VSP), noting that the vehicles often operated under low-load or near-idle conditions.

In this context, vehicular congestion is strongly linked to instantaneous fuel consumption [19,20,21], so quantifying this parameter in cities worldwide is a tool for decision making. In Ecuador, small municipalities like Sangolquí lack basic information about vehicular traffic conditions and road fuel consumption. Hence, using methodological tools according to the local environment is key for the effective implementation of public policies through knowledge supported by empirical evidence.

This study proposes a comparative analysis of fuel efficiency between extreme traffic conditions in high congestion hours and regular vehicular flow periods. This comparison allows us to quantify how different traffic regimes affect fuel consumption, identifying specific patterns associated with each condition and focusing on the main routes in the historic center of Sangolquí, Ecuador. In Sangolquí, particularly in its historic center and commercial areas, a marked contrast in vehicle behavior is observed when comparing peak hours (07:00–09:00, 13:00–15:00, and 17:00–19:00) with off-peak hours. During these congestion peaks, approximately 19,086 vehicles pass through the canton’s main intersections [6].

Information is obtained through the OBD-II onboard diagnostic system to develop the work. Parameters such as absolute intake manifold pressure (MAP), revolutions per minute (RPM), vehicle speed (VSS), torque, and power are monitored [22,23,24]. The selected gear is identified using these values according to automatic learning algorithms. Using the longitudinal dynamics of the car plus the knowledge of the selected gear, the vehicle’s consumption in traffic and free flow conditions is estimated to show the differences found.

This article is structured as follows: Section 2 describes the methodology used for the development, ranging from vehicle selection, data acquisition, and information post-processing. Section 3 uses machine learning techniques to discretize the vehicle gears and identify the ideal algorithm for this scenario. In addition, the difference in fuel consumption according to vehicle congestion in the city is described. In Section 4, the differences found between the two studies are explained. Finally, conclusions and future work are shown in Section 5.

## 2. Materials and Methods

### 2.1. Data Collection and Analysis

**Figure 1:**
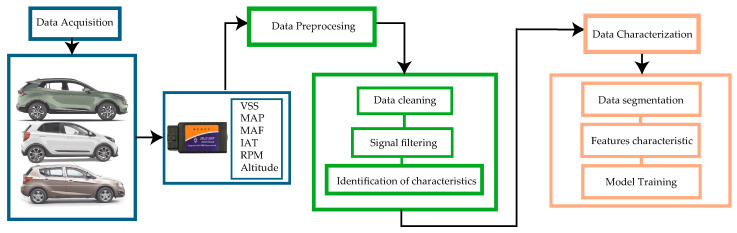
Methodology and procedure proposed.

The flowchart shown in Figure 1 represents the methodology employed in this research organized into three main stages: data acquisition, preprocessing, and characterization.

The process starts with data acquisition, using an ELM327 recorder (Shenzhen Eload Electronic Technology Co., Ltd., Shenzhen, China) connected to the OBD-II port to capture vehicle operating variables such as vehicle speed (VSS), engine speed (RPM), manifold absolute pressure (MAP), mass airflow (MAF), and intake air temperature (IAT). Altitude is obtained using GPS data to contextualize the analysis under driving conditions at 2500 m above sea level.

Data preprocessing is performed as follows. The PID signals were filtered using a centered 5 s moving average window, while the altitude signal was smoothed using a Savitzky–Golay filter [25]. Outliers were removed through interpolation based on an IQR-based detection method with a threshold factor of 1.5.

Finally, the processed data were segmented to extract relevant features such as accelerations and gear shifts. K-means clustering was used to define gear shift thresholds. A neural network model was trained to relate vehicle speed (VSS) and engine load (RPM) with gear selection, enabling the characterization of driving profiles. Multiple machine learning algorithms were tested for gear classification, with Fine KNN achieving the highest accuracy. A multiple linear regression model was also developed to estimate fuel consumption based on predicted gear and other variables.

Vehicle data for the Chevrolet Sail, Kia Sportage, and Kia Picanto models were obtained using a data logger based on the ELM327 interface through the OBD-II connector, which allowed for the real-time storage of the ECU (Electronic Control Unit) sensor variables (see Table 1). The sampling frequency was 1 Hz [26]. The type of connection could be via Bluetooth or Wi-Fi, efficiently capturing and storing relevant data from the road.

**Figure 2:**
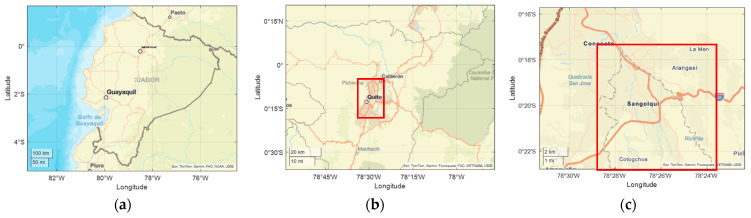
Geographical location: (**a**) Ecuador; (**b**) Pichincha; (**c**) Sangolquí.

This study was conducted in Sangolquí, located in Pichincha, Ecuador. The area is situated at an average altitude of 2500 m above sea level, with relatively stable environmental conditions, an average temperature of 17 °C, and no precipitation during the tests. A representation of the study location can be seen in Figure 2. Sangolquí has a population of 85,852 inhabitants according to the 2022 census [27]. The data provided are then cleaned, segmented, and analyzed using K-means clustering to identify patterns in gear efficiency and longitudinal vehicle dynamics. This analysis provides insights into the relationship between driving behavior, gear selection, and overall vehicle performance, which play a significant role in understanding energy consumption.

### 2.2. Category of M1 Vehicles

**Figure 3:**
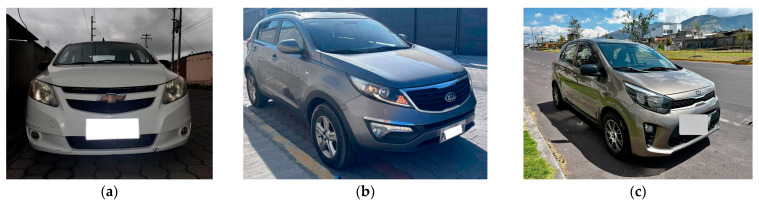
Selected vehicles: (**a**) Chevrolet Sail; (**b**) Kia Sportage; (**c**) Kia Picanto.

M1 vehicles are those designed to transport passengers with a maximum capacity of eight passengers, not including the driver. These daily-use vehicles are ideal for personal use, providing comfort and safety in urban and suburban environments [28]. Technically, M1 vehicles are not limited to size or weight, but their design aims to meet daily transportation needs. They can have various propulsion systems, such as internal combustion engines (diesel or gasoline), hybrid, electric, and even new technologies like hydrogen fuel cells. The vehicles used for extracting PID signals were established according to the engine displacement ranges set by Ecuadorian Technical Standard 2656 [28], which are vehicles ≤ 1000cc (e.g., Kia Picanto 1000cc), 1000cc < vehicles ≤ 1600cc (e.g., Chevrolet Sail 1400cc), and 1600cc < vehicles ≤ 2000cc (e.g., Kia Sportage 2000cc). The technical specifications of the vehicle and their representation are presented in Table 2 and Figure 3.

To evaluate the impact of traffic on fuel consumption and vehicle energy efficiency, tests were conducted in two distinct scenarios: peak hours (with traffic congestion) and off-peak hours (without congestion). Table 3 and Table 4 present the schedules and routes of the three vehicles used in the study during these periods. The selected vehicles—a Chevrolet Sail, a KIA Sportage R, and a KIA Picanto—were monitored on different days and times to capture a variety of driving conditions.

### 2.3. Longitudinal Vehicle Dynamics

**Figure 4:**
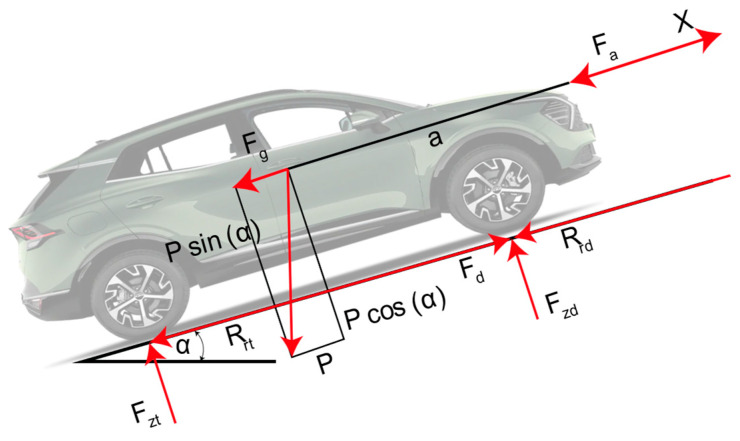
Longitudinal vehicle dynamics.

A vehicle encounters various forces that must be overcome for movement, including aerodynamic resistance, rolling resistance, slope resistance, and inertia, as illustrated in Figure 4. From the dynamics of the car, these forces can be calculated, allowing for the estimation of energy consumption. The tractive force $F_{t}$ is related to aerodynamic $F_{a}$ and rolling resistance $R_{x}$, as well as inertial forces $F_{i}$ and slope resistance $R_{slope}$ [29].

(1) $$F_{t}=F_{a}+R_{x}+R_{g}+F_{i}$$

When an object moves over a surface, the opposition it encounters is known as rolling resistance, which is primarily affected by the deformation of the contacting surfaces. The resistance force acts in the opposite direction to the movement and is directly proportional to the normal force applied to the tire’s contact patch. Where *R_x_* is the rolling resistance [N], *f_r_* is the coefficient of rolling resistance [dimensionless], *m* is the vehicle’s mass [kg], *g* is the gravity [m/s^2^], and *θ* is the slope [degrees].

(2) $$Fx=mgf_{r}\cos\left(\theta \right)$$

Aerodynamic forces affect the vehicle by creating effects such as drag, lateral forces, and moments of inertia, which can result in rotations like pitch, roll, or yaw. Where *Fa* is the drag force [N], *ρ* is the air density [kg/m^3^], *A_f_* is the frontal area of the vehicle [m^2^], *C_x_* is the drag coefficient of the vehicle [dimensionless], and *V* is the vehicle’s linear speed [m/s].

(3) $$Fa=\frac{1}{2}\rho {{C_{x}A_{f}V}_{GPS\;i}}^{2}$$

**Figure 5:**
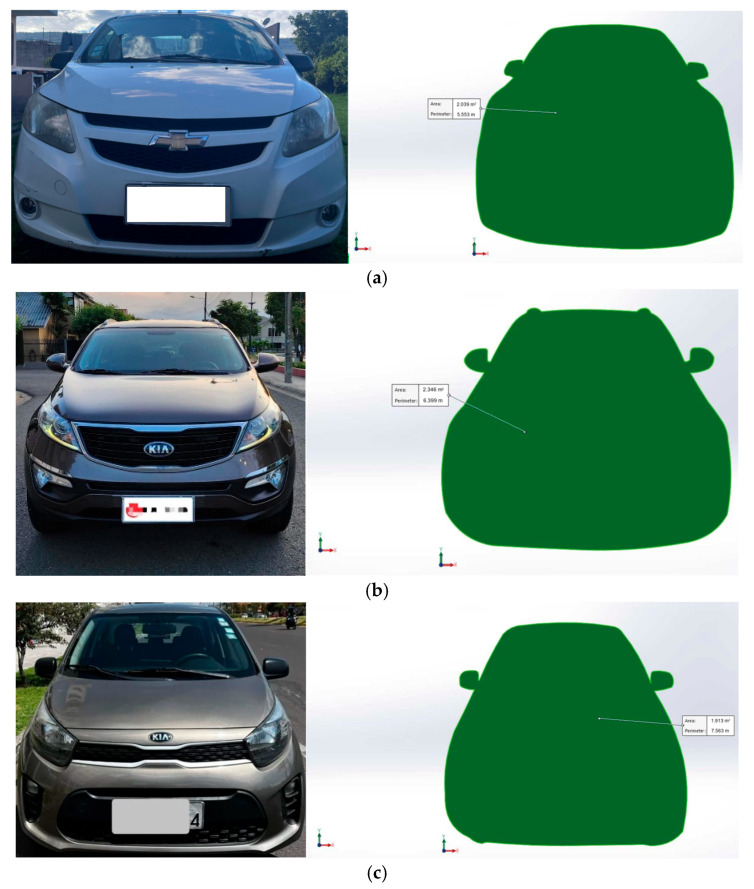
Front area. (**a**) Chevrolet Sail; (**b**) Kia Sportage; (**c**) Kia Picanto.

Parameters such as the frontal area of the vehicle are calculated by creating a cross-section in a computer-aided design program, as shown below in Figure 5. The dimensions are obtained from the manufacturer’s website, and the results for the frontal area are presented in Table 5.

**Figure 6:**
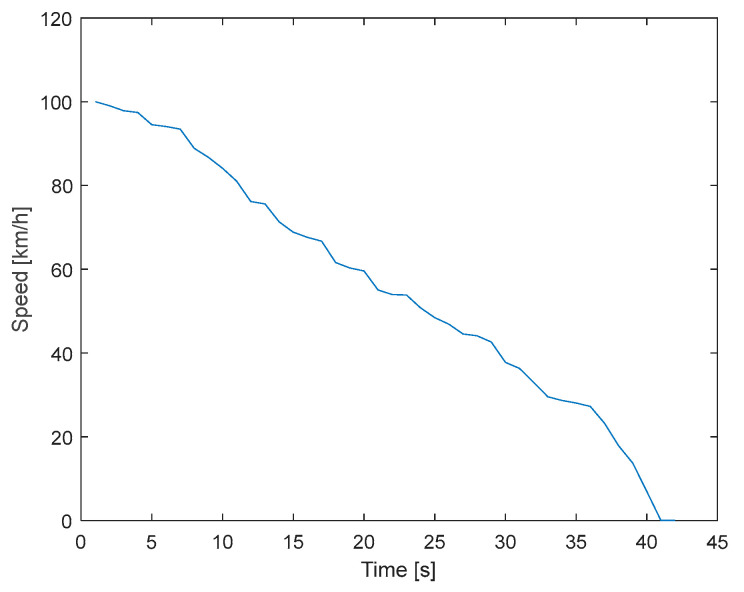
Chevrolet Sail coast-down test.

The coast-down method or deceleration test allows for the determination of a vehicle’s rolling resistance coefficients (*f_r_*) and the aerodynamic coefficient (*C_x_*). This resistance is calculated based on the deceleration recorded when the car reaches a 100 km/h speed and rolls freely in neutral until it comes to a complete stop, as presented in Figure 6. When on a flat surface, the force due to the slope is negated, and by putting the transmission in neutral, the engine is disconnected from the gearbox, eliminating inertial forces. These data are collected using an application called GPS Logger [30].

(4) $$\frac{dVmi}{dt}=\frac{-{0.5CdpaAV}_{ei-1}-{frgcos\theta }_{ei-1}-{mgsen\theta }_{ei-1}\;}{M}$$

Ref. [31] propose a speed minimization method in which the coefficients *Cd* and *Fr* are determined as those values that minimize the absolute differences between the measured speed *V* and the estimated speed $\hat{V}$ at all time points *t* during the coast-down test. This minimization is formulated as a nonlinear programming problem, where the objective function is to minimize. Various numerical methods exist to solve this nonlinear problem, with the reduced gradient method (GRG) standing out for its simplicity.

(5) $$Minimize=\;\sum {\left(v_{\left(t\right)}-{\hat{v}}_{\left(t\right)}\right)}_{\left(Cd,f_{r}\right)}^{2}\;$$

The slope resistance is calculated using the altitude obtained through GPS, which reflects the ascent during the route. The slope force is the resistance experienced when ascending or descending a slope.

(6) $$F_{slope}=mg\sin\left(\frac{{Alt}_{\;i+1}-{Alt}_{\;i}}{S_{i+1}-S_{i}}\right)$$

The inertial force results from the product of the vehicle’s mass and its longitudinal acceleration ($F_{i}=m\;a$), where the longitudinal acceleration $a_{x}$ is calculated using the GPS velocity as a reference.

(7) $$a_{x}i=\frac{V_{GPS\;i+1}-V_{GPS\;i}}{t_{i+1}-t_{i}}$$

Force results from the sum of the forces required to overcome the vehicle’s movement. The torque at the wheel directly depends on the dynamic radius of the wheel, which is determined by the following factors:

(8) T=Ft·rn [Nm]

where:

*T* = torque [Nm];
*Ft* = wheel force [N];
*rn* = effective radius [m].

**Figure 7:**
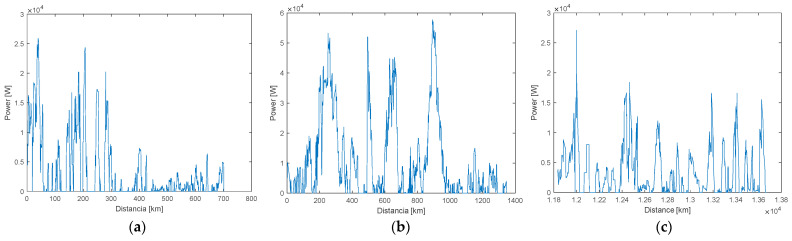
Power obtained from Chevrolet Sail: (**a**) morning; (**b**) afternoon; (**c**) evening and night.

One of the variables used in torque calculation is employed to calculate wheel power: the total force on the wheels, which is multiplied by the vehicle’s speed. A representation of the power output for the Chevrolet Sail is illustrated in Figure 7, showing the three phases of the day. Moreover, the speed recorded through PID signals from the data logger in km/h must be converted to meters per second (m/s) to improve calculation accuracy. The wheel power of the three analyzed vehicles was calculated using the following equation.

(9) Pr=Ft·V [kW]

**Figure 8:**
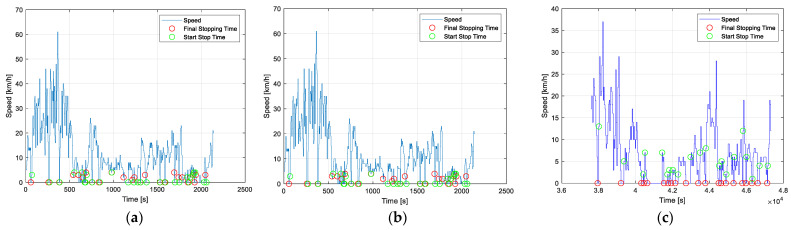
Stopping times: (**a**) morning; (**b**) afternoon; (**c**) evening and night.

In Figure 8, the stopping times of each vehicle on its respective route are analyzed, allowing for the examination of movement patterns and the evolution of distance over time. Significant variations in route conditions are identified through the vehicles’ speed profiles. Additionally, time series help detect periods of inactivity, such as stops due to traffic or traffic lights, by showing changes in the monitored parameters, indicating transitions between activity and pause.

## 3. Results

### 3.1. Estimation of the Performance of the Selected Gear

**Figure 9:**
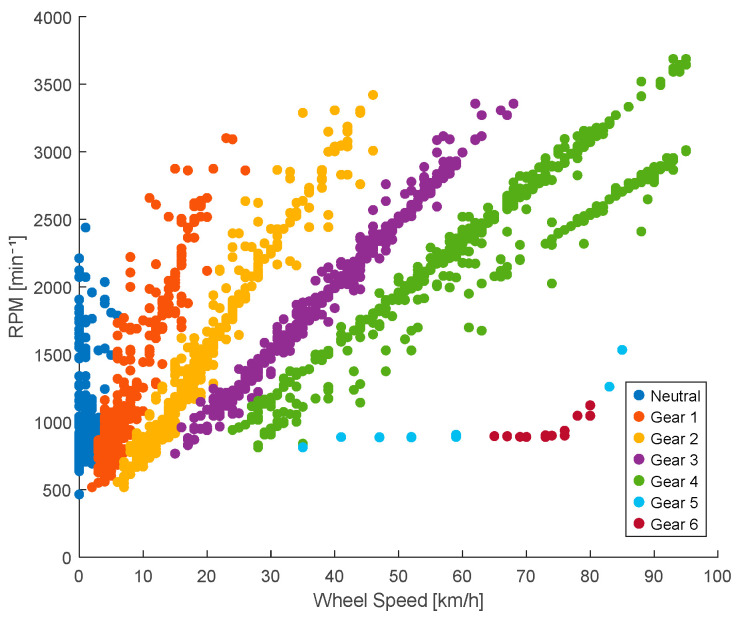
K-means classification.

The relationship between the vehicle speed and engine speed is given by $r_{i}=\frac{{VSS}_{i}}{{RPM}_{i}}$. In this example, corresponding to the Kia Sportage, the K-means algorithm allows the grouping of the points generated in the speed diagram using the signals obtained from the Vehicle Speed Sensor (VSS) and the engine revolutions (RPM) [32]. This unsupervised machine learning algorithm partitions the data into k distinct clusters based on feature similarity, minimizing variance within each cluster. As illustrated in Figure 9, each gear is visually represented by a different color, allowing for the intuitive identification of the clusters. These groupings correspond to the number of gears in the vehicle plus the neutral position; for example, if the vehicle has five forward gears, six clusters are formed.

Several models were trained and evaluated to determine the best classifier for this estimation model. The models included decision trees (with Fine, Medium, and Coarse options), K-Nearest Neighbors (KNN) with various configurations (Fine, Medium, Coarse, Weighted, Cosine, and Cubic), logistic regression, and a linear Support Vector Machine (SVM). Among all the models assessed, the KNN (Fine KNN) model was chosen as the optimal classifier due to its outstanding performance and generalization capabilities. This model achieved an accuracy of 99.7%, the highest among all models evaluated, and an error rate of just 0.3%, the lowest for the validation set. The metrics for the trained classification models are detailed in Table 6.

Table 7 shows the confusion matrix of the KNN model (Fine KNN) used for the classification of gears in the KIA Sportage, including neutral (class 1) and six gears (classes 2 to 7). In this matrix, the rows represent the actual classes, and the columns represent the predicted classes, reflecting the distribution of the model predictions. Values on the main diagonal correspond to correct predictions (true positives), while values outside the diagonal indicate classification errors, known as false positives and false negatives.

**Figure 10:**
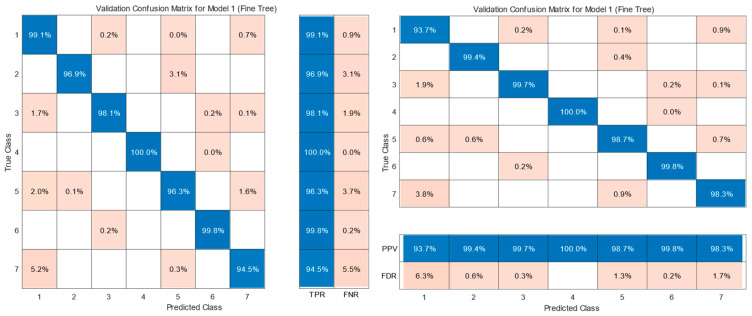
KNN (Fine KNN) classification: true class vs. predicted class correct and incorrect classification.

Figure 10 shows the performance percentages of the KNN model (Fine KNN) in gear shift classification. In part (a), the true positive rate (TPR) and false negative rate (FNR) are presented, where the model exhibits a TPR higher than 99.4% in all gears and an FNR lower than 0.6%, with the perfect identification of gear 2 (100% TPR). Part (b) shows the positive predictive value (PPV) and false discovery rate (FDR), highlighting the accuracy and reliability of the model in gear classification, with a low incidence of errors.

**Figure 11:**
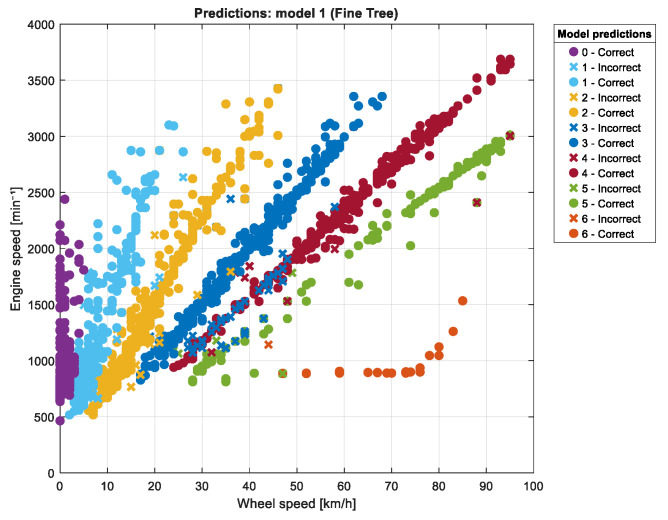
KNN (Fine KNN) classification of gear states in a Kia Sportage (0: neutral, 1–6: gears). The classification model predicts gear states labeled as 0 to 6, where 0 corresponds to neutral and 1 to 6 correspond to gears 1 to 6, respectively.

Gear selection is initially estimated based on the VSS and RPM signals obtained through the OBD system. While gear ratios in a given vehicle are fixed, relying solely on the VSS/RPM ratio can result in inaccurate gear identification under real-world urban driving conditions. To address this, a supervised learning algorithm was implemented to establish a relationship between the observed signals and the longitudinal dynamics of the vehicle. The input feature vector was defined as IN = [VSS, RPM, Ax, S, Fr, Fg, Fa, P, T], where each component corresponds to kinematic or dynamic properties influencing gear selection. The output variable represents the selected gear, obtained through the K-means algorithm, denoted as OUT = [0, 1, 2, 3, 4, 5, 6]. Figure 11 illustrates the clustering results, clearly separating gear states based on the input features. For vehicles with five forward gears plus neutral, the output dimension was adjusted accordingly to OUT = [0, 1, 2, 3, 4, 5].

A model based on a fitting neural network with a single hidden layer composed of 10 neurons was implemented to complement the classification. The training function used was Levenberg–Marquardt, which was selected for its ability to facilitate fast convergence. For the training, validation, and testing of the model, data splitting in proportions of 70%, 15%, and 15%, respectively, was used. This configuration enabled the optimization of the model’s performance and the evaluation of its generalization capacity.

Precision is a metric that quantifies the proportion of correct optimistic predictions the model makes. In this case, a value of 99.9% indicates that nearly all instances classified as positive truly belong to the correct class, highlighting the model’s reliability in identifying gear shifts. Precision is computed using the following equation, where TP represents true positives and FP represents false positives.

(10) $${Precision}_{i}=\frac{TP}{TP+FP}=0.990$$

On the other hand, recall measures the model’s ability to identify positive instances within the dataset correctly. With a value of 99.7%, the model effectively captures most positive cases, minimizing the omission of actual gear shifts. Recall is calculated using the following equation, where TP represents true positives and FN false negatives.

(11) $${Recall}_{i}=\frac{TP}{TP+FN}=0.997$$

The F1-score quantifies the balance between these metrics, representing the harmonic mean of precision and recall. In this case, a value of 99.8% was obtained, indicating that the model achieves both a high rate of correct predictions and adequate coverage of actual instances. The F1-score is calculated as follows.

(12) $$F1\text{-}Score=\frac{2\;*\;Precision\;*\;Recall}{Precision+Recall}=0.998$$

Two different machine learning techniques with complementary functions were used in this study. First, the K-means algorithm, an unsupervised clustering method, was applied to group gear shift points based on the relationship between engine speed (RPM) and vehicle speed (VSS). In this way, it was possible to identify gear clusters without needing labeled data, which is particularly useful when no direct gear information is available in the dataset. These clusters, representing the different gait levels, including neutral, served as pseudo-labels for the next stage.

These labels were then used to train a supervised classifier. Among the models evaluated, the Fine KNN algorithm demonstrated the best performance in classifying gear changes using a broader input vector that includes RPM and VSS and parameters related to longitudinal dynamics such as acceleration, road slope, and resistive forces. This two-stage process—unsupervised grouping followed by supervised classification—ensures that gear identification is data-driven and context-aware, allowing for generalization to unknown driving conditions.

### 3.2. Calculating Fuel Consumption

Fuel consumption can be estimated from the signal obtained from the intake manifold using the ideal gas law [33,34,35]. In this relationship, *P* represents the absolute pressure in the intake manifold in kPa, *V* is the cylinder volume in m^3^, *R* is the ideal gas constant, *n* indicates the number of moles, and *T* is the ambient temperature obtained from the intake air temperature (IAT) sensor reading of the engine:

(13) P·V=n·R·T

(14) $$ṁ=\frac{P\cdot V}{R\cdot T}\cdot M_{air}\cdot n_{vol}\cdot \frac{RPM}{2\cdot 60}$$

where:

*V* = cylinder volume [cm^3^];
ṁ = air mass flow [kg/s];
RPM = engine speed.

The fuel flow is determined based on the air–fuel ratio, dividing the estimated air mass flow by the stoichiometric mixture of 14.7:1 and the density of 737 g/L for gasoline, as shown in Equation (5). With these calculated parameters, it is possible to estimate the fuel consumption in liters per hour (L/h).

(15) $$\dot{V}\;=\frac{ṁ}{AFR\cdot \rho_{fuel}}$$

where

ARF = air–fuel ratio [dimensionless];
$\rho_{fuel}$ = fuel density [kg/m^3^];

### 3.3. Consumption and Speed as a Function of Driving in Traffic

**Figure 12:**
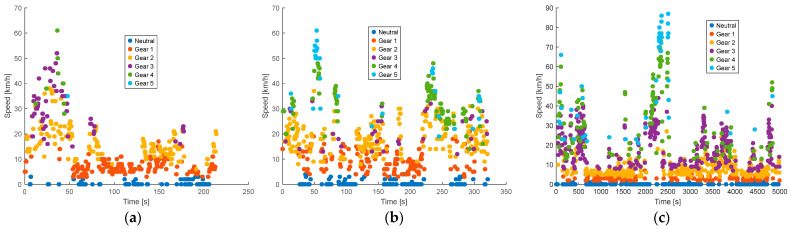
Classification of Chevrolet Sail gears according to vehicle speed with traffic: (**a**) morning; (**b**) afternoon; (**c**) evening and night.

Gear selection is graded according to vehicle speed for the Chevrolet Sail, which has five forward gears plus neutral. In the morning and afternoon, fourth and fifth gears are typically engaged at speeds above 40 km/h, while in the evening, most gear selections occur within the 10–20 km/h range. First and second gears are consistently concentrated at speeds below 20 km/h across all periods. The dispersion of the points in the morning and afternoon suggests frequent transitions between gears, reflecting stop-and-go traffic conditions. In contrast, evening data show a narrower speed range and fewer transitions, indicating more stable driving. These patterns are visually represented in Figure 12, which illustrates the distribution of gear usage by vehicle speed across different time bands.

**Figure 13:**
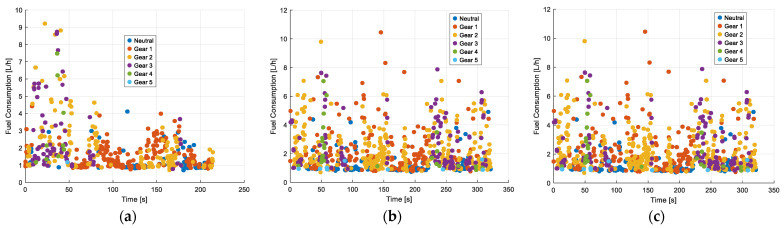
Classification of Chevrolet Sail gears according to fuel consumption with traffic: (**a**) morning; (**b**) afternoon; (**c**) evening and night.

The relationship between fuel consumption (L/h) and gear shifts is analyzed. Consumption values range from 0 to 12 L/h, with higher records in first and second gear, indicating greater fuel demand under low-speed and acceleration conditions. In the morning and afternoon, consumption is distributed across all gears, with peak values close to 10 L/h during periods of higher engine load. At night, consumption remains below 6 L/h in most cases, suggesting lower power and acceleration demands during this period. Additionally, when idle times occur, the vehicle is generally kept neutral, meaning no power is generated, but fuel consumption tends to increase due to engine idling. Figure 13 illustrates these consumption patterns across different gears and time bands, highlighting the variability in demand throughout the day.

**Figure 14:**
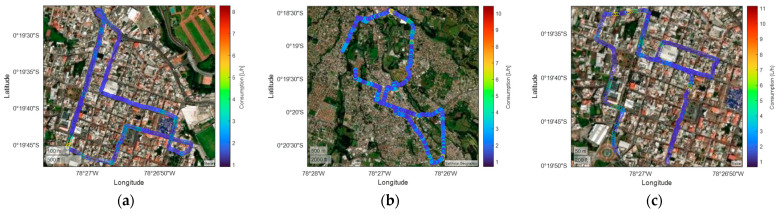
Characterization of Chevrolet Sail fuel consumption with traffic: (**a**) morning; (**b**) afternoon; (**c**) evening and night.

The spatial distribution of fuel consumption throughout the route is examined for morning, afternoon, and night scenarios. As shown in Figure 14, high consumption zones—typically near intersections and areas requiring acceleration—display values close to 10 L/h, particularly during daytime hours. Demand is more consistent at night and remains mostly under 6 L/h, with occasional spikes caused by slope or abrupt speed changes. These variations highlight the influence of driving dynamics and road topography, with critical points located along España, Alfaro, Bolívar, and General Enríquez streets.

### 3.4. Fuel Efficiency and Speed in Gears with No Traffic

**Figure 15:**
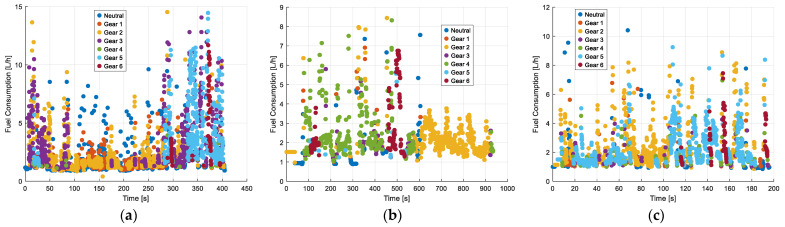
Classification of Kia Sportage gears according to fuel consumption with no traffic: (**a**) morning; (**b**) afternoon; (**c**) evening and night.

Figure 15 presents the classification of fuel consumption based on the gear engagement of the Kia Sportage, which is equipped with six forward gears plus neutral, during different periods of the day with no traffic. In the morning (a), lower gears (first and second) dominate, with fuel consumption peaking at 15 L/h, while higher gears (fourth, fifth, and sixth) are used less frequently. In the afternoon (b), fuel consumption is more evenly distributed across all gears, with a maximum consumption of around 9 L/h, suggesting a more stable driving pattern. At night (c), most data points correspond to the first, second, and third gears, with fuel consumption rarely exceeding 10 L/h. The dispersion of points is greater in the morning and afternoon, indicating frequent gear changes and variations in acceleration, which lead to higher fuel demand.

**Figure 16:**
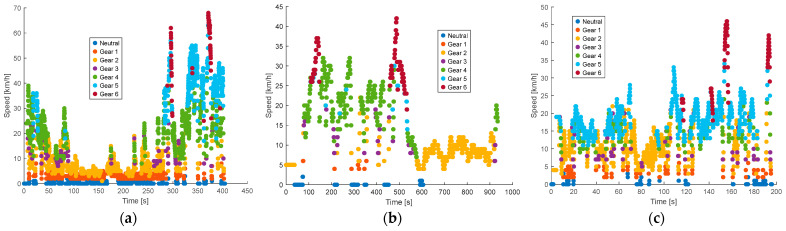
Classification of Kia Sportage gears according to vehicle speed with no traffic: (**a**) morning; (**b**) afternoon; (**c**) evening and night.

The gear rankings of the Kia Sportage are presented as a function of vehicle speed under no traffic conditions during the morning, afternoon, and evening periods. As illustrated in Figure 16, during the morning, the higher gears (fourth, fifth, and sixth) are engaged at speeds above 50 km/h, reflecting relatively fluid driving conditions. In contrast, the afternoon period shows a more varied distribution of gear usage, with the fourth gear appearing from 40 km/h onwards. Most of the data are concentrated below 30 km/h at night, with a clear predominance of the first three gears, indicating lower average speeds and potentially more stop-and-go conditions.

**Figure 17:**
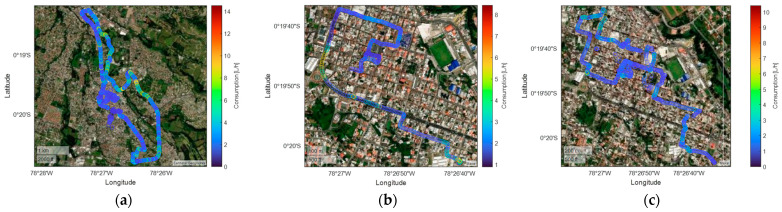
Characterization of Kia Sportage fuel consumption with no traffic: (**a**) morning; (**b**) afternoon; (**c**) evening and night.

Figure 17 presents the spatial characterization of fuel consumption on the Kia Sportage no traffic route in the three periods analyzed. In the morning, the points of highest consumption are located in sectors of acceleration and slopes, with records above 12 L/h in certain areas. In the afternoon, the consumption is more distributed along the route, with maximum values close to 10 L/h. At night, fuel consumption shows less variability, with most records below 8 L/h. The lower fuel demand at night suggests more stable driving conditions and less acceleration requirements.

### 3.5. Regression Model for Instantaneous Fuel Consumption Estimation

From the classification of gears using the Fine KNN model, the estimated gear information was used as an explanatory variable to develop a multiple linear regression model. This model aims to estimate the instantaneous fuel consumption in L/100 km from vehicle operating variables recorded by OBD-II sensors during urban trips in peak and non-peak traffic conditions. The explanatory variables consider vehicle speed (VSS), engine revolutions per minute (RPM), intake manifold pressure (MAP), longitudinal acceleration (Ax), and gear engagement.

The mathematical expression of the resulting model is as follows:

(16) $$\begin{array}{l} Fuel\;Consumption\;(L/100\;km)\\ \;=\;20.86-0.0910\times VSS+0.00089\times RPM+0.3277\times MAP+0.1743\times Ax\\ \;-7.6751\times {Gear}_{1}-16.33\times {Gear}_{2}-19.89\times {Gear}_{3}-22.05\times {Gear}_{4}-20.05\times {Gear}_{5} \end{array}$$

where ${Gear}_{i}$ represents indicator variables for gears 1 to 5 and neutral gear is considered the base category. The model was fitted with 66,489 valid observations, reaching a coefficient of determination R^2^ = 0.897, indicating a high explanatory capacity. Table 8 summarizes the model coefficients as well as their interpretation and statistical significance. In addition, the root mean square error (RMS) is 2.06, a relatively low value suggesting a good model fit.

Interpreting the model coefficients allows us to understand the individual effect of each variable on fuel consumption. For example, vehicle speed (VSS) has a negative coefficient, indicating that the higher the speed, the lower the instantaneous fuel consumption due to more constant and efficient driving. Engine revolutions (RPM) and manifold pressure (MAP) show a positive impact, showing that a higher demand for the engine increases consumption. Acceleration also has a direct effect, with an estimated increase of 0.174 L/100 km per unit for longitudinal acceleration.

As for the gears, the negative coefficients of the indicator variables (gear1 to gear5) reflect significantly lower consumption compared to the neutral position, which is used as the base category. The highest gears (3, 4, and 5) show the most negative coefficients, suggesting higher efficiency in these operating conditions.

**Figure 18:**
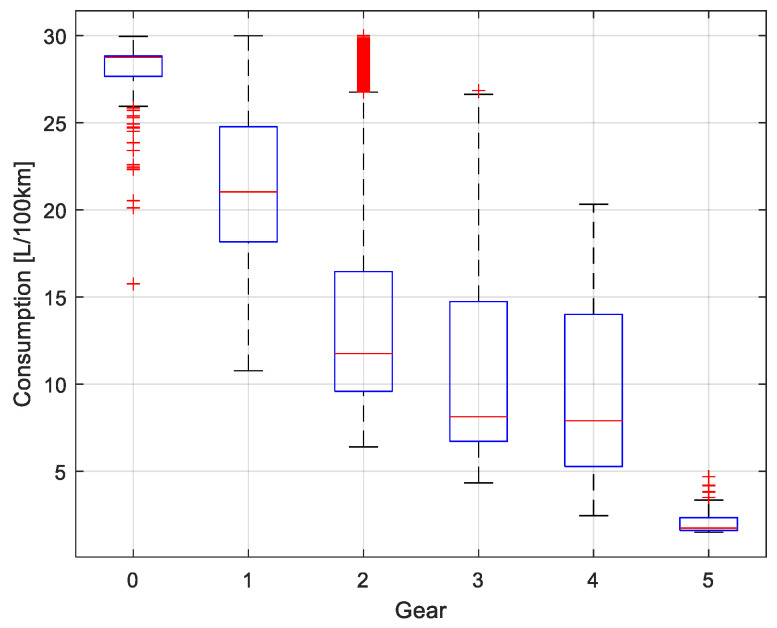
Model consumption per run of the vehicle fleet in peak and off-peak hours.

Figure 18 shows a boxplot illustrating the distribution of estimated instantaneous fuel consumption in L/100 km as a function of the engaged gear, where gear 0 corresponds to neutral. Fuel consumption in neutral is the highest and most dispersed, with numerous outliers attributed to prolonged idling during stopped traffic. As higher gears are engaged, consumption decreases progressively, with gears 3, 4, and 5 showing greater efficiency, which is reflected in lower medians and reduced variability. In contrast, lower gears (1 and 2) display higher variability and elevated consumption levels. This behavior is consistent with the fact that the model was trained using data collected during peak and off-peak traffic periods. During peak traffic conditions, the frequent use of lower gears—associated with repeated stops and accelerations—leads to significantly increased fuel consumption.

### 3.6. Evaluation of Fuel Consumption and Stopping Time Under Different Conditions: With and Without Traffic

**Figure 19:**
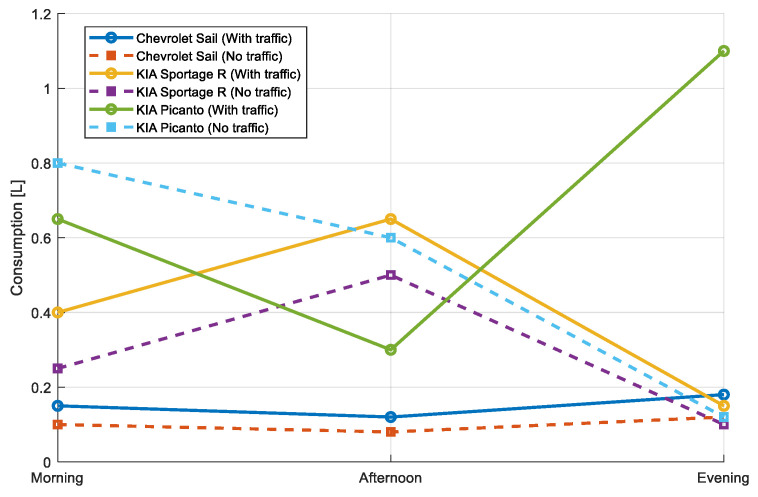
Evaluation of fuel consumption in the morning, afternoon, and evening.

Fuel consumption varies significantly between traffic and non-traffic conditions for the vehicles analyzed. As shown in Figure 19, in the case of the Chevrolet Sail, consumption with traffic is 0.15 L (morning), 0.12 L (afternoon), and 0.18 L (evening), while in no traffic it is reduced to 0.10 L, 0.08 L, and 0.12 L, respectively. This pattern reflects an average 50% increase in consumption due to traffic. For the KIA Sportage R, consumption with traffic reaches 0.65 L in the afternoon, which is the most demanding period, while under no traffic conditions it is reduced to 0.50 L, showing an improvement of 23%. The KIA Picanto presents high consumption at night with traffic (1.10 L), which is drastically reduced to 0.12 L without traffic, reflecting an improvement of 89%. As a result, traffic increases fuel consumption, especially during peak hours such as the evening, and smaller vehicles are more sensitive to these variations.

**Figure 20:**
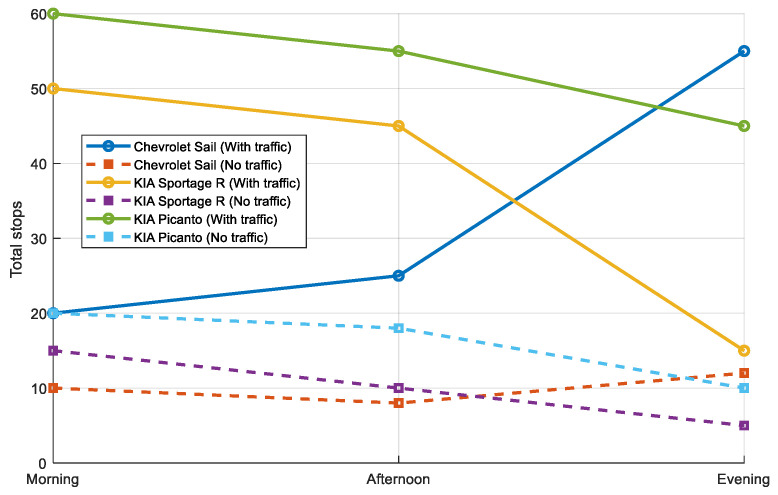
Evaluation of morning, afternoon, and evening downtime.

Stopping times also show notable differences between traffic and non-traffic conditions. As illustrated in Figure 20, for the Chevrolet Sail, the total stops during traffic are 20 (morning), 25 (afternoon), and 55 (evening). In contrast, under non-traffic conditions, these values are reduced to 10, 8, and 12, respectively, indicating an average increase of 300% due to traffic. Similarly, for the KIA Sportage R, stopping times during traffic are 50 (morning), 45 (afternoon), and 15 (evening), which decrease to 15, 10, and 5 under non-traffic conditions, reflecting an increase of 233%. Finally, the KIA Picanto exhibits stopping times of 60 (morning), 55 (afternoon), and 45 (evening) during traffic, dropping to 20, 18, and 10 without traffic, corresponding to a 200% increase. These results demonstrate that traffic significantly prolongs stopping times, especially in the morning and afternoon, with the evening being the period with the least impact.

## 4. Discussion

This study presents a novel approach to predicting driving style and traffic conditions by integrating On-Board Diagnostics II (OBD II), using artificial neural networks (ANNs) as the core model. Unlike previous studies that often rely on a single data source or focus on isolated prediction tasks, our model combines multiple signal types—including speed, engine RPM, and longitudinal dynamics data in real-world conditions—thus improving the robustness of the prediction in varied driving contexts.

The study “Driving Style and Traffic Prediction with Artificial Neural Networks Using On-Board Diagnostics and Smartphone Sensors” proposes an approach based on artificial neural networks (ANNs) to predict driving style and traffic conditions using OBD II data and smartphone sensors. This work reports remarkable metrics, such as an accuracy of 0.86, recall of 0.81, accuracy of 0.92, and F1-score of 0.83 in driving style prediction [36].

Vasavi et al. present a study on gear prediction in manual transmission vehicles using artificial neural networks (ANNs). The model uses engine speed (RPM) and vehicle speed (km/h) as inputs and gear as output. The results show a generalization mean square error of 0.005, indicating high accuracy [37].

Tollner et al. propose road-type classification models, mainly using vehicle speed data obtained through the OBD-II standard in vehicles with internal combustion engines and hybrids. For supervised learning with road type labels (city, rural, and highway), the study metrics highlight a maximum accuracy of 96.21% achieved by the combination of cross-validation and loss function weighting; the neural network base model already outperformed the conventional method, achieving an accuracy of 93.25% versus 89.9% for the latter [38].

In our study, the combination of unsupervised K-means and supervised clustering (KNN fine) proved effective when actual gear labels were not directly available. K-means was used to infer gear clusters based on signal behavior, and KNN leveraged this information along with additional vehicle dynamics parameters to classify gear states with high consistency. This hybrid approach increased the reliability of the model and its practical applicability in real-world conditions, especially when labeled datasets are sparse.

However, several limitations were identified during model development. One of the main problems was the limited generalizability of the ANN-based gear prediction model to different types of vehicles. Although the architecture shows good performance, the hyperparameters varied considerably from vehicle to vehicle, implying the need to adjust the model for each vehicle, which limits scalability. In addition, the model’s reliance on accurate RPM and speed measurements introduces sensitivity to sensor noise and possible errors caused by mechanical vibrations or road surface irregularities.

Despite these limitations, the proposed method shows promising potential for integration into advanced driver assistance systems (ADAS). The model’s ability to infer gear state under real driving conditions lays the groundwork for improving gear change timing based on dynamic context, such as terrain, traffic, and driving behavior. This integration could contribute to more efficient fuel usage and enhanced vehicle performance, especially in semi-automated or driver feedback systems.

## 5. Conclusions

This study presents a gear shift classification and fuel consumption analysis algorithm based on processing PID (parameter identification) signals obtained through the OBD II system. By acquiring and analyzing real-time data such as revolutions per minute (RPM), mass airflow (MAF), wheel speed (VSS), and intake air temperature (IAT), the factors influencing fuel consumption in M1 category vehicles under usual driving conditions in high traffic areas were identified.

The results show that, at low speeds and under frequent stops, the engine consumes fuel without generating useful power, which hurts the vehicle’s energy efficiency. This phenomenon is particularly evident during peak traffic hours, where long stopping times and frequent gear changes significantly increase fuel consumption.

Using the K-means algorithm, the relationship between vehicle speed (VSS) and revolutions per minute (RPM) was used to establish gear shift patterns. However, it was determined that this ratio alone is insufficient to establish the driver’s gear choice, as it depends on multiple parameters, such as longitudinal vehicle dynamics, driving conditions, and other external factors. To overcome this limitation, a supervised learning algorithm, KNN (Fine KNN), was implemented, which established a relationship between the vehicle characteristics, represented by the input vector IN = [VSS, RPM, Ax, S, Fr, Fg, Fa, P, T], and the gear selection, represented by the output vector OUT = [0, 1, 2, 3, 4, 5, 6], where each value corresponded to a specific gear.

The algorithm developed, based on the KNN (Fine KNN) model, showed an accuracy of 99.7% in the classification of gear changes, reaching an accuracy of 99.7%, an error rate of 0.3%, a precision of 99.8%, a recall of 99.7%, and an F1-score of 99.8%, showing the high reliability of the metrics presented given a robust classification model.

Likewise, the gear estimated by the KNN model was integrated as an explanatory variable in a multiple linear regression model to estimate instantaneous fuel consumption in L/100 km. This model, fitted with more than 66,000 valid observations, achieved a coefficient of determination R^2^ of 0.897 and a root mean square error (RMS) of 2.06, suggesting a good fit. The interpretation of the coefficients showed that higher gears (3, 4, and 5) are associated with lower fuel consumption. At the same time, neutral has the highest level of consumption and dispersion, especially during prolonged stops in peak traffic conditions.

Sangolquí is an intermediate-scale city with a population of 85,852 inhabitants according to the 2022 census. Its altitude of 2500 m above sea level and irregular topography may influence driving patterns, limiting its representativeness for larger urban environments or those with different geographic characteristics. Therefore, it is necessary to incorporate data from other cities with variability in their demographic and geographic conditions to develop more robust and generalizable models, allowing for their application in diverse urban scenarios.

Based on the results, this algorithm shows potential for integration into driver assistance systems (ADAS) to optimize gear shifting and enhance energy efficiency in real time. Future research may focus on its application in autonomous vehicles to improve real-time decision-making processes.

## Figures and Tables

**Table 1:** Registration of the route variables.

Variables	Nomenclature	Range	Units
Vehicle Speed Sensor	VSS	0–110	km/h
Engine Speed	RPM	0–6000	min^−1^
Engine Coolant Temperature	ECT	0–92	°C
Intake Air Temperature	IAT	8–42	°C
Manifold Absolute Pressure	MAP	16–75	kPa
Mass Air Flow	MAF	0–35	g/s
Throttle Position Sensor	TPS	0–100	%

**Table 2:** Specifications of selected vehicles.

Vehicle	Brand	Model	Year	Odometer [km]	Vehicle Weight [kg]	Engine Displacement [cm^3^]	Torque [Nm]	Power [kW]	Number of Gears
Vehicle 1	KIA	Picanto	2018	115,349	886	998	141@4850 rpm	69@6300 rpm	5 + Reverse
Vehicle 2	Chevrolet	Sail	2013	315,622	1087	1400	131@4200 rpm	76@6000 rpm	5 + Reverse
Vehicle 3	KIA	Sportage R	2020	98,458	1490	2000	191@4700 rpm	112@6200 rpm	6 + Reverse

**Table 3:** Schedule of vehicle routes during peak traffic hours.

Route	Vehicle	Date	Start Time	End Time
Vehicle 1	Chevrolet Sail	Tuesday 05/11/2024	13:18:52	14:12:24
		Thursday 07/11/2024	17:37:10	19:00:22
		Friday 03/01/2025	09:12:28	09:48:12
Vehicle 2	KIA Sportage R	Monday 30/12/2024	09:32:26	10:13:23
		Monday 30/12/204	13:48:44	14:48:13
		Monday 30/12/2024	17:50:11	19:12:57
Vehicle 3	KIA Picanto	Friday 15/11/2024	13:39:26	14:17:14
		Friday 03/12/2024	08:48:04	10:08:36
		Monday 30/12/2024	18:58:08	19:34:21

**Table 4:** Vehicle routing schedule during non-traffic hours.

Route	Vehicle	Date	Start Time	End Time
Vehicle 1	Chevrolet Sail	Monday 10/02/2025	11:03:59	11:27:18
		Friday 28/02/2025	13:42:43	14:19:20
		Friday 28/02/2025	16:37:23	17:10:06
Vehicle 2	KIA Sportage R	Monday 17/02/2025	15:52:16	16:07:51
		Wednesday 26/02/2025	19:35:00	20:07:38
		Thursday 27/02/2025	10:35:13	11:42:40
Vehicle 3	KIA Picanto	Monday 03/03/2025	20:11:35	20:25:37
		Wednesday 05/03/2025	15:32:41	15:58:21
		Friday 07/03/2025	09:54:22	10:21:57

**Table 5:** Frontal area of test vehicles.

Vehicle	Brand	Model	Height [mm]	Width [mm]	Frontal Area [mm^2^]	Frontal Area [m^2^]
Vehicle 1	KIA	Picanto	1595	1495	1,912,864.54	1.913
Vehicle 2	Chevrolet	Sail	1503	1690	2,039,072.22	2.039
Vehicle 3	KIA	Sportage R	1645	1855	2,346,431.97	2.346

**Table 6:** Classification model performance metrics.

Model	Accuracy Validation (%)	Error Rate Validation (%)	Precision Avg (%)	Recall Avg (%)	F1-Score Avg (%)	Training Time (s)	Prediction Speed (obs/s)
1 Tree (Fine Tree)	98.5	1.5	98.6	98.5	98.5	15.340	~85,000
2 Tree (Medium Tree)	86.8	13.2	87.0	86.8	86.9	12.890	~90,000
3 Tree (Coarse Tree)	70.7	29.3	71.0	70.7	70.8	10.560	~95,000
4 KNN (Fine KNN)	99.7	0.3	99.8	99.7	99.8	27.245	~78,000
5 KNN (Medium KNN)	98.1	1.9	98.2	98.1	98.1	25.780	~76,000
6 KNN (Coarse KNN)	87.7	12.3	87.9	87.7	87.8	20.450	~80,000
7 KNN (Cosine KNN)	98.2	1.8	98.3	98.2	98.2	28.340	~77,000
8 KNN (Cubic KNN)	97.9	2.1	98.0	97.9	97.9	29.120	~75,000
9 KNN(Weighted KNN)	99.6	0.4	99.7	99.6	99.7	30.120	~75,000
10 Logistic Regression	72.0	28.0	72.5	72.0	72.2	8.230	~100,000
11 Efficient Linear SVM	93.7	6.3	93.8	93.7	93.7	18.560	~88,000

**Table 7:** Validation confusion matrix.

True Class	1	2	3	4	5	6	7
1	4150	0	6	1	0	0	2
2	0	159	0	0	0	0	0
3	1	0	5033	0	0	7	0
4	0	0	0	5303	0	15	0
5	0	0	0	0	1315	0	3
6	0	0	10	20	0	5234	0
7	10	0	0	0	1	0	3208

**Table 8:** Model coefficients of test vehicles.

Variable	Estimate	SE	tStat	Interpretation
Intercept	20.86	0.17236	121.12	Estimated baseline consumption when all explanatory variables are zero.
VSS	−0.0910	0.0020152	−44.787	For each additional 1 km/h, consumption decreases by 0.0910 L/100 km.
RPM	0.00089	3.8004 × 10^−5^	22.957	For each additional 1 RPM, consumption increases by 0.00089 L/100 km.
MAP	0.3277	0.0006457	507.34	For each additional 1 kPa in manifold pressure (MAP), consumption increases by 0.3277.
Ax	0.1743	0.027113	6.6654	Accelerations increase consumption by 0.1743 L/100 km per unit of measurement.
Gear_1_	−7.6751	0.16822	−45.641	Using gear 1 reduces 7.68 L/100 km compared to the base category.
Gear_2_	−16.33	0.16853	−96.937	Gear 2: −16.33 L/100 km compared to neutral.
Gear_3_	−19.89	0.17245	−115.44	Gear 3: −19.89 L/100 km compared to neutral.
Gear_4_	−22.05	0.18252	−120.95	Gear 4: −22.05 L/100 km compared to neutral.
Gear_5_	−20.05	0.2575	−79.055	Gear 5: −20.05 L/100 km compared to neutral.

## Data Availability

The original contributions presented in the study are included in the article; further inquiries can be directed to the corresponding author.

## Abbreviations

The following abbreviations are used in this manuscript:

*Af*	Front area
*Alt*	Altitude
*ANN*	Artificial neural network
*a_x_*	Longitudinal acceleration
*C_X_*	Drag coefficient
ρ	Density
*Fa*	Aerodynamic resistance
*Fg*	Gravitational resistance
*Fr*	Rolling resistance force
*Fslope*	Force slope
*GPS*	Global position system
IAT	Air intake temperature
*Lat*	Latitude
*Lon*	Longitude
*m.a.s.l*	Meters above sea level
*m*	Mass
*OBD*	On-board diagnostics
*PID*	Parameter of identification
*P*	Power
*RDE*	Real driving emissions
RPM	Engine speed
*T*	Torque
V	Volume
